# Genome analysis of multidrug resistant *Enterococcus faecium* and *Enterococcus faecalis* circulating among hospitalized patients in uMgungundlovu District, KwaZulu-Natal, South Africa

**DOI:** 10.1186/s12879-024-09380-3

**Published:** 2024-07-04

**Authors:** Raspail Carrel Founou, Luria Leslie Founou, Mushal Allam, Arshad Ismail, Sabiha Yusuf Essack

**Affiliations:** 1 Antimicrobial Research Unit, School of Health Sciences, University of KwaZulu-Natal, Durban, 4000 South Africa; 2 Department of Microbiology-Haematology and Immunology, Faculty of Medicine and Pharmaceutical Sciences, University of Dschang, Dschang, Cameroon; 3 Antimicrobial Resistance and Infectious Disease Research Unit, Research Institute of Centre of Expertise and Biological Diagnostic of Cameroon (CEDBCAM-RI), Yaoundé, Cameroon; 4 Reproductive, Maternal, Newborn and Child Health (ReMARCH) Research Unit, Research Institute of the Centre of Expertise and Biological Diagnostic of Cameroon (CEDBCAM-RI), Yaoundé, Cameroon; 5 Bioinformatics & Applied Machine Learning Research Unit, EDEN Biosciences Research Institute (EBRI), EDEN Foundation, Yaoundé, Cameroon; 6 Sequencing Core Facility, National Institute for Communicable Diseases a Division of the National Health Laboratory Service, Johannesburg, 2131 South Africa; 7 Department of Biochemistry and Microbiology, Faculty of Science, Engineering and Agriculture, University of Venda, Thohoyandou, 0950 South Africa; 8 Institute for Water and Wastewater Technology, Durban University of Technology, Durban, 4000 South Africa

**Keywords:** WGS, *Enterococcus*, Mobilome, Hospitals, Carriage

## Abstract

**Background:**

Vancomycin-resistant enterococci (VRE) are important pathogens categorized as high-priority bacteria in the Global Priority List of Antibiotic-Resistant Bacteria to Guide Research, Discovery, and Development of New Antibiotics published by the World Health Organization. The aim of this study was to determine the risk factors, resistance, virulence, mobilomes associated with multidrug-resistant and clonal lineages of Enterococcus faecium and faecalis circulating among hospitalized patients following the health system in South Africa, using whole genome sequencing (WGS).

**Methods:**

A cross-sectional study was conducted during a two-month periods among hospitalized patients in 2017. Rectal swabs were collected from patients admitted to medical and surgical wards in an urban tertiary hospital, and a rural district hospital in uMgungundlovu district, South Africa. Enterococci were screened for vancomycin resistance on bile esculin azide agar supplemented with 6 mg/L of vancomycin and confirmation of VRE was done using ROSCO kits. Conventional and real-time PCR methods were used to ascertain the presence of VanA, VanB, VanC-2/3 and VanC-1 genes. All six multidrug-resistant Enterococcus faecalis and faecium selected were identified using multiplexed paired-end libraries (2 × 300 bp) with the Nextera XT DNA sample preparation kit (Illumina, San Diego, CA, USA) and genome sequencing was done using Illumina MiSeq instrument with 100× coverage at the National Institute of Communicable Diseases Sequencing Core Facility, South Africa. Antibiotic resistance genes, virulence factors, plasmids, integrons and CRISPR were characterized using RAST, ResFinder, VirulenceFinder, PlasmidFinder, PHAST and ISFinder respectively.

**Results:**

Sequencing analysis revealed that these strains harbouring numerous resistance genes to glycopeptides (*van*C[100%], *vex*3[100%], *vex*2[83,33%] and *van*G[16,66%]), macrolides, lincosamides, sterptogramine B (*erm*B[33,32%], *Isa*[16,66%], *eme*A[16,66%]) and tetracyclines (*tet*M[33,32%]) in both district and tertiary hospitals. Multidrug efflux pumps including MATE, MFS and pmrA conferring resistance to several classes of antibiotics were also identified. The main transposable elements observed were in the Tn3 family, specifically Tn1546. Four single sequence types (STs) were identified among E. faecium in the district hospital, namely ST822, ST636, ST97 along with a novel ST assigned ST1386, while one lineage, ST29 was detected in the tertiary hospital.

**Conclusion:**

The study reveals the genetic diversity and high pathogenicity of multidrug-resistant Enterococcus faecalis and faecium circulating among hospitalized patients. It underlines the necessity to implement routine screening of admitted patients coupled with infection control procedures, antimicrobial stewardship and awareness should be strengthened to prevent and/or contain the carriage and spread of multidrug resistant *E. faecium* and *E. faecalis* in hospitals and communities in South Africa.

**Supplementary Information:**

The online version contains supplementary material available at 10.1186/s12879-024-09380-3.

## Introduction

*Enterococcus spp.* are Gram-positive cocci, frequently isolated in the gastrointestinal tract of both humans and animals [1, 2]. This genus encompasses more than 40 species, but only *E. faecalis, E. faecium* and *E. avium* have been identified as clinically important due to their implications in serious difficult-to-treat nosocomial infections such as endocarditis, urinary tract infections, peritonitis, bacteraemia, neonatal sepsis, meningitis, surgical wound and intra-abdominal infections in hospitals and communities [1, 2].

Enterococci are clinically relevant because of the (i) emergence of vancomycin-resistant *E. faecium* (VRE), (ii) high-levels of resistance to multiple antibiotics, (iii) transfer of resistance gene from VRE to *Staphylococcus aureus*, (iv) presence of different selective pressures increasing the proliferation and rapid spread of VRE, (v) few therapeutic options for disease management, and (vi) limited success of VRE containment measures [1, 3–5]. Leclerc et al., (1988) described nine operons capable of conferring resistance to glycopeptides [6]. The differentiation of these operons is based on ligase genes encoding D-alanyl-D-lactate ligase (VanA, VanB, VanD, and VanM) or D-alanyl-D-serine ligases (VanC-1, VanC-2, VanC-3, VanE, VanG, VanN and VanL) [7]. VRE were recently ranked as high priority in the Global Priority List of Antibiotic-Resistant Bacteria to Guide Research, Discovery and Development of New Antibiotics by the World Health Organization (WHO) [8].

Van A phenotype strains of *E. faecium* were first detected from clinical cases of VRE infections in Europe in 1986, where they were associated with outbreaks in hospitals, particularly in patients with severe underlying diseases or an immunocompromised status [1, 7]. The overuse of glycopeptides and extended-spectrum cephalosporins in hospital settings has probably contributed to the increased prevalence and spread of these resistant pathogens [9]. In Africa, the first cases of VRE infections were described in South Africa where a 10.9% prevalence of VRE-colonized patients was reported at a hospital in 1997 although this threat is relatively under-investigated in the country [8].

This study therefore assessed the carriage, risk factors, resistance and virulence genes associated with multidrug resistant *Enterococcus faecium* and *faecalis* isolated from hospitalized patients in uMgungundlovu District, KwaZulu-Natal, South Africa.

**Methods**.

### Study population and settings

This study was conducted in two healthcare facilities, a 505-bed tertiary hospital in an urban area and a 141-bed district hospital in rural area from May to June 2017. The district hospital (H1) cover four services i.e., obstetrics and gynaecology, paediatrics and child health, general surgery and general medicine with 141 beds. The tertiary hospital (H2) offers several specialties, receives referral patients according to a nationally agreed referral plan and has approximately 505 beds.

### Patient enrolment and questionnaire data collection

After explanation of the study, oral and written informed consent was obtained from all participants. Patients thereafter completed a questionnaire that yielded socio-demographic information while the clinical history was extracted from patient records. Information was codified prior to analysis to ensure confidentiality.

### Sample collection

Sample collection took place in both surgical and general medical wards. Rectal swabs were aseptically collected with sterile cotton swabs in Amies transport media from all admitted in-patients > 18 years old, at admission, after 48 h and at discharge whenever possible.

### Culture and identification

Rectal swabs (*n* = 45 specimens) were cultured onto Bile-Esculin-Azide agar (Oxoid, Dardilly, France) with and without vancomycin (6 mg/L). After incubation for 18–24 h at 37 °C, each black colony growing on Bile-Esculin-Azide agar supplemented with vancomycin (BEA + VAN) that further hydrolysed and reduced 0.04% potassium tellurite, was selected for Gram staining, the oxidase and catalase tests and L-pyrrolidonyl-b-naphthylamidase activity. Biochemical identification was confirmed using API Strept (bioMérieux, Marcy l’Etoile, France). Pure colonies of *E. faecium* and *E. faecalis* were stored into tryptone soya broth (TSB) (Merck, Darmstadt, Germany) supplemented with 20% glycerol at − 20 °C for future use.

### Phenotypic screening

All colonies were phenotypically screened for vancomycin, teicoplanin and daptomycin resistance using the package of MRSA, VISA, GISA, VRE ROSCO DIAGNOSTICA Kit (Taastrup, Denmark) using 0.5 McFarland on Mueller-Hinton agar (Oxoid, Dardilly, France) according to the manufacturer’s instructions.

### Antimicrobial susceptibility testing by microbroth dilution

Minimum inhibitory concentrations (MICs) were determined by broth microdilution. Ampicillin, cefoxitin, gentamycin, streptomycin, ciprofloxacin, moxifloxacin, erythromycin, clindamycin, linezolid, teicoplanin, vancomycin, tetracycline, doxycycline, tigecycline, fusidic acid, trimethoprim, nitrofurantoin, and chloramphenicol, were tested and interpreted according to the European Committee on Antimicrobial Susceptibility Testing (EUCAST, 2017) breakpoints using *E. faecium* ATCC 29,212 as the control strain.

### Genomic extraction and purification

Genomic DNA of selected strains were extracted using the GenElute Bacterial Genomic DNA Kit (Sigma-Aldrich, St. Louis, MO, USA) according to the manufacturer’s instructions. DNA were stored at -20^°^C. The concentration and purity of the extracted gDNA were determined by fluorometric analysis (Qubit®) and agarose gel electrophoresis, respectively.

### Conventional polymerase chain reaction (PCR)

All confirmed VRE were screened by simplex PCR to identify associated vancomycin resistance genes with specific primers for VanA, VanB, and VanC2/3 as previously described (Supplementary Table 1) [10]. The oligonucleotide primers were also synthesized by Inqaba Biotech (Pretoria, South Africa). PCR were performed in 0.2 ml PCR-tube in a programmable BioRad Thermal Cycler (CA, Foster City, USA) with the following conditions: initial denaturation at 95 °C for 4 min, 30 cycles of denaturation at 95 °C for 30 s, annealing at 56.5 °C for 1 min, and elongation at 72 °C for 1 min followed by a final extension at 72 °C for 7 min and an infinite hold a 4 °C. The generated amplicons were resolved by horizontal electrophoresis on 1.5% (wt/vol) Tris-Borate-EDTA (Merck, Darmstadt, Germany) agarose gels together with the Quick-load®1-kb (Biolabs, New England, France) and run in an electric field of 110 V for 2 h 30 min. Electrophoresis gels were visualized by a UV light trans-illuminator (BioRad Laboratories, CA, Foster City, USA), images were captured using a Gel Doc™ XR + system (BioRad Laboratories, CA, Foster City, USA) and analysed by Image Lab™ Software (version 4.0, BioRad Laboratories, CA, Foster City, USA).

### Real-time polymerase chain reaction (RT-PCR)

RT- PCR was performed to ascertain specific vancomycin resistance genes on a programmable automate QuantStudio5™ (Applied Biosystems, CA, USA) using the Taqman Universal Master Mix 2× (Applied Biosystems, CA, USA) and ready-made assays (Thermo Scientific, CA, USA). Thermal temperature running conditions were as follows: UNG activation at 50 °C for 2 min, initial denaturation at 95 °C for 10 min, 30 cycles of denaturation 95 °C for 10 s, annealing/extension at 60 °C for 1 min and a final extension at 60 °C for 30 s. The results were interpreted with QuantStudio™ design and analysis software version 1.4 (Applied Biosystems, CA, USA).

### Genome sequencing and phylogenetic analysis

**Fig. 1:**
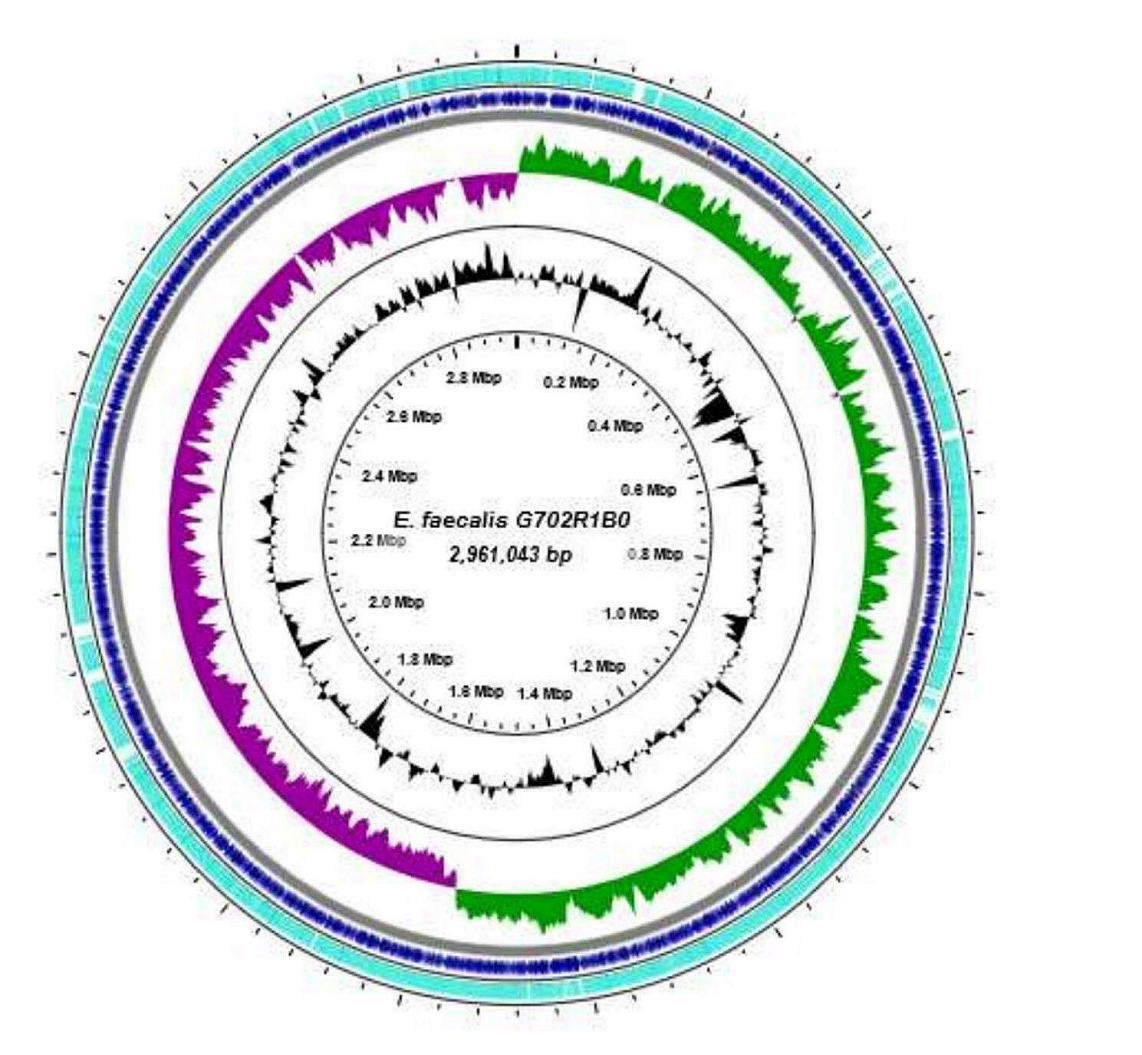
*E. faecalis*G702R1B0 ring representation using CGView Server version 1.0 (Grant et Sothard, 2008). The inner ring shows the percent of identity comparing *E. faecalis* G702R1B0 and the finished genome of *E. faecalis* DENG1 (CP004081.1). The first two inner rings show the GC content and GC skew. The next inner ring, alternating blue and green regions represents the contigs delimitation of G702R1B0. The last outer ring presents the genome of *E. faecalis* G702R1B0

The Nextera XT DNA sample preparation kit (Illumina, San Diego, CA, USA) was used for the preparation of multiplex paired-end libraries (2 × 300 bp). The Illumina MiSeq machine was used for library sequencing with 100× coverage. The generated reads were checked for quality and trimmed using the CLC Genomics Workbench version 10 (CLC, Bio-QIAGEN, Aarhus, Denmark). *De novo* assembling was subsequently performed with CLC Genomics and SPAdes version 3.5.0 [11]. The assembled reads were uploaded and annotated using the Bacterial Analysis Pipeline of GoSeqIt tools, NCBI PGAP (https://www.ncbi.nlm.nih.gov/genome/annotation_prok/) and ARG-ANNOT (http://en.mediterranee-infection.com). ResFinder [12], VirulenceFinder [13] ISfinder [14] (Siguier, Perochon, Lestrade, Mahillon, & Chandler, 2006), PlasmidFinder [15] Phaster, and CRISPRsFinder were used for the identification of antibiotic resistance genes, virulence factors, insertion sequences, plasmids, bacteriophages and CRISPRs respectively. The multi-locus sequence type (MLST) was determined from the WGS data. Contigs of *E. faecalis* G702R1B0 were mapped against the finished genome of *E. faecalis* DENG1 (CP004081.1) for visualization of the genomic structure (Fig. 1) as described [16]. Phylogenetic analyses were performed to contextualize our strains against a collection from international complete genomes (accession no.: CP004081; NC017316; NC004668; CP003351; NC017960; CP019988) (Fig. 1). The core genes were determined from the annotated genome assemblies, predicted coding regions were extracted and converted into protein sequences. A phylogeny was drawn for *E. faecalis and faecium* using Rapid large-scale prokaryote pangenome analysis (Roary; https://sanger-pathogens.github.io/Roary/) to estimate the tree for the core genome.

### Data analysis

Data was coded and entered into an Excel spreadsheet (Microsoft Office 2016) and analysed using STATA (version 14.0, STATA Corporation, TX, USA). Risk factors for VRE colonization were ascertained by univariate and multivariate logistic regression analysis. Prevalence of VRE carriage was compared between categories (viz. hospital, ward and time-point) using the chi square test, and a p-value < 0.05 was regarded as statistically significant.

## Results

### Study population and risk factors for VRE in carriage

Out of 72 hospitalized patients contacted, 45 (60%) agreed to participate, answered the questionnaire and were sampled. Overall, males were more colonized than females at admission and at discharge (Table 1). Similarly, thirty-one (69%) patients were treated with an antibiotic during their hospital stay with the mean age being 50.7 years (range 19–70 years). Patients in the district hospital were more likely to be colonized by VRE bacteria at admission (44%), after 48 h (64%) and discharge (100%) than those at the tertiary hospital (Table 1). Gender, antibiotics use, co-morbidity, previous hospitalization, transfer from another hospital were the main risk factors identified at admission in both hospitals while the odds of VRE colonization were higher in surgical wards after 48 h (Tables 2 and 3).

**Table 1:** Faecal carriage of multidrug resistant Enterococcus spp. isolated from hospitalized patients in relation to socio-demographic factors, clinical history and diagnosis at admission in a rural district, and an urban tertiary hospital. Out of the 45 patients enrolled, some refused rectal sampling after 48 h and/or at discharge, while some were discharged or transferred after 48 h, leading to variability in number

Variables	District Rural Hospital *n* = 27			Tertiary Urban Hospital *n* = 18		
	Admission, (%)	After 48 h, (%)	At discharge, (%)	Admission, (%)	After 48 h, (%)	At discharge, (%)
Overall	44	64	100	39	43	50
Gender						
Female	36	67	100	50	0	0
Male	64	60	0	56	60	100
Previous hospitalization (within one year)						
**Yes**	50	50	100	75	33	0
No	47	67	100	44	50	50
Antibiotic use (during hospital stay)						
**Yes**	50	50	100	67	0	0
**No**	47	70	100	50	75	50
Referral from another hospital						
**Yes**	100	0	0	50	33	0
No	46	64	100	57	40	50
Hospital ward						
Medicine	53	56	100	60	40	0
Surgery	40	80	100	50	50	100

**Table 2:** Univariate logistic regression of potential risk factors for VRE carriage in the district and tertiary hospital

Variables	District hospital		Tertiary Hospital	
	Admission OR (95% CI)	After 48 h OR (95% CI)	Admission OR (95% CI)	After 48 h OR (95% CI)
Gender (F or M)	3.15 (0.61–16.31)	0.75 (0.08–7.21)	1.25 (0.11–13.24)	1
Antibiotic use (Yes or No)	1.13 (0.20–6.04)	0.43 (0.04–4.64)	2 (0.13–29.80)	1
Previous hospitalization	1.11 (0.17–6.97)	0.50 (0.02–10.25)	3.75 (0.27–51.37)	0.5 (0.02–11.08)
Transferred from another hospital (Yes or No)	1	1	0.75 (0.08–6.71)	0.5 (0.02–11.08)
Hospital Ward (Medicine or Surgery)	0.58 (0.11–2.95)	3.2 (0.25–41.21)	0.66 (0.07–6.40)	1.25 (0.05–40.63)
Hospital (Rural District or Urban Tertiary)	1.26 (0.33–4.84)	0.42 (0.06–2.66)	0.90 (0.14–5.71)	0.35 (0.37–14.65)

**Table 3:** Multivariate logistic regression of predictors of multidrug resistant Enterococci carriage at admission in the district and tertiary hospital

Variables	District hospital OR (95% CI)	Tertiary Hospital; OR (95% CI)
Gender (F or M)	4.44 (0.59–33.21)	1.19 (0.09–14.69)
Previous hospitalization (Yes or No)	1.87 (0.09–36.58)	3.41 (0.14–81.94)
Current Antibiotic use (Yes or No)	1.46 (0.07–27.66)	1.05 (0.03–32.62)
Referral from another hospital	1	0.95 (0.07–12.83)
Hospital ward (Medicine or Surgery)	0.4 (0.05–2.97)	0.76 (0.05–10.05)

Altogether, 24 (53%) patients were asymptomatic faecal carriers of VRE with some harbouring multiple strains, yielding a total of 38 non-duplicate VRE in both hospitals. Of these 15 (39%) and 23 (61%) were confirmed *E. faecium* and *E. faecalis*, respectively. More specifically, 14 (54%) *E. faecium* and 12 (46%) *E. faecalis* were isolated in the district hospital while 11 (92%) *E. faecalis* and one (8%) *E. faecium* were identified in the tertiary hospital (Table 4).

**Table 4:** Antibiotic resistance profiles of multidrug resistant *E. faecium* and *E. faecalis* isolated from hospitalized patients

Antibiotics	District hospital, *n* = 26				Tertiary hospital, *n* = 12			
	*E. faecalis*(*n* = 12)		*E. faecium*(*n* = 14)		*E. faecalis*(*n* = 11)		*E. faecium*(*n* = 1)	
	MIC (µg/ml) range	No. resistant isolates (%)	MIC (µg/ml) range	No. resistant isolates (%)	MIC (µg/ml) range	No. resistant isolates (%)	MIC (µg/ml) range	No. resistant isolates (%)
Ampicillin	4-≥512	8 (67)	0.5–256	6 (43)	1-≥512	3 (27)	8	0 (0)
Cefoxitin	32-≥512	12 (100)	128-≥512	14 (100)	16-≥512	11 (100)	≥ 512	1 (100)
Erythromycin	4-≥512	12 (100)	2-≥512	14 (100)	1-≥512	10 (91)	0.5	0 (0)
Clindamycin	2-≥512	12 (100)	8-≥512	14 (100)	4-≥512	11 (100)	128	1 (100)
Teicoplanin	4-≥512	12 (100)	2-≥512	14 (100)	4-≥512	11 (100)	32	1 (100)
Vancomycin	8-≥512	10 (83)	8-≥512	10 (71)	8-≥512	10 (91)	32	1 (100)
Tigecycline	8-128	7 (58)	2–64	10 (71)	8-≥512	10 (91)	16	1 (100)
Fusidic acid	64-≥512	10 (83)	16-≥512	10 (83)	2-256	9 (82)	256	1 (100)
HLR*-Gentamicin	128-≥512	8 (67)	32-≥512	9 (64)	8-≥512	7 (64)	64	0 (0)
HLR-Streptomycin	256-≥512	8 (67)	64-≥512	9 (64)	16-≥512	10 (91)	256	1 (100)
Chloramphenicol	64-≥512	10 (83)	64-≥512	10 (71)	64-≥512	10 (91)	128	1 (100)
Ciprofloxacin	128-≥512	10 (83)	128-≥512	10 (71)	64-≥512	10 (91)	128	1 (100)
Moxifloxacin	16-≥512	10 (83)	8-≥512	10 (71)	2-≥512	10 (91)	2	0 (0)
Doxycycline	16–256	10 (83)	0.5–256	9 (64)	2-512	9 (82)	4	1 (100)
Tetracycline	32-≥512	9 (75)	4-≥512	10 (71)	4-≥512	10 (91)	$16$	1 (100)
Nitrofurantoin	32-≥512	9 (75)	64-≥512	10 (71)	64-≥512	10 (91)	128	1 (100)
Trimethoprim	≥ 512	11 (92)	128-≥512	14 (100)	≥ 512	11 (100)	≥ 512	1 (100)

*HLR: High-Level resistance.

### Antimicrobial susceptibility and resistance determinants

High levels of antibiotic resistance were observed among isolates in both the district and tertiary hospitals. In the district hospital, 100% resistance to cefoxitin, erythromycin, clindamycin, teicoplanin was evident in both bacterial species while 100% resistance was observed against all antibiotics except moxifloxacin, gentamicin, erythromycin, and ampicillin in *E. faecium* in the tertiary hospital (Table 4).

VanC1 was the only glycopeptide resistant gene detected in all VRE isolates. In addition, the *E. faecalis* ST6 (G702R1B0) carried *Van*G together with the vancomycin tolerance locus (*vex2*, *vex3*), macrolide, lincosamide, sterptogramines B (*erm*B, *Isa, emeA*), tetracycline (*Tet*M), fosfomycine (*fos*B) and fluoroquinolones (*Par*C, *Par*E, *gyr*A, *gyr*B) resistance genes. All *E. faecium* and *E. faecalis* harboured *Vex*2 and *Vex*3 alongside with the multidrug resistant efflux pumps (MATE, MFS, *pmr*A) encoding for resistance to several antibiotic classes (Tables 5 and 6).

**Table 5:** Resistance to selected antibiotics resistant *E. faecium* and *E. faecalis* isolated from single patients

Patient ID	Isolate	Hospital	Ward	Time-points	REP Cluster	Antibiotics (MIC µg/ml)											
						VAN	TEI	E	CLI	CN	STR	CIP	MOX	FA	DOX	TET	FT
A100	*E. faecalis* (A100R1B0)	District	Medicine	Admission	/	16	8	8	≥ 512	256	≥ 512	≥ 512	512	256	128	256	256
	*E. faecalis* (A100R2B0)			After 48 h	/	8	8	8	≥ 512	128	≥ 512	≥ 512	≥ 512	256	128	≥ 512	128
	*E. faecalis* (A100R3B0)			Discharge	B1	16	32	≥ 512	≥ 512	512	≥ 512	≥ 512	≥ 512	256	128	≥ 512	128
A101	*E. faecalis* (A101R1B0)	District	Medicine	Admission	B2	32	256	≥ 512	128	512	≥ 512	≥ 512	256	256	128	≥ 512	256
A105	*E. faecalis* (A105R1B0)	District	Medicine	Admission	B3	16	8	8	256	256	≥ 512	256	≥ 512	256	16	32	≥ 512
	*E. faecalis* (A105R2B0)			After 48 h	B1	16	8	8	256	≥ 512	≥ 512	≥ 512	≥ 512	64	16	32	≥ 512
A107	*E. faecalis* A107R1B0	District	Medicine	Admission	B2	≥ 512	8	16	256	128	≥ 512	≥ 512	≥ 512	128	128	≥ 512	32
	*E. faecalis* A107R2B0			After 48 h	/	16	4	4	256	128	≥ 512	≥ 512	≥ 512	128	256	512	≥ 512
A108	*E. faecium* A108R1B0C1	District	Medicine	Admission	A4	16	4	16	≥ 512	128	256	128	128	128	8	64	128
	*E. faecium* A108R2B0			After 48 h	A2	16	8	2	512	32	512	≥ 512	256	128	16	16	64
A109	*E. faecium* A109R1B0	District	Medicine	Admission	/	16	8	16	8	128	256	128	128	32	64	512	512
	*E. faecium* A109R2B0			After 48 h	A1	≥ 512	8	32	32	≥ 512	≥ 512	≥ 512	128	16	4	16	≥ 512
	*E. faecium* A109R3B0			Discharge	/	≥ 512	8	8	32	≥ 512	≥ 512	≥ 512	≥ 512	16	4	16	≥ 512
A110	*E. faecium* (A110R1B0)	District	Medicine	Admission	/	16	8	≥ 512	≥ 512	≥ 512	≥ 512	≥ 512	≥ 512	32	256	≥ 512	256
A113	*E. faecalis* (A113R1B0)	District	Medicine	Admission	Singleton 1	16	8	4	256	128	512	128	128	64	128	512	128
A200	*E. faecalis* (A200R3B0)	District	Surgery	Discharge	B1	≥ 512	≥ 512	≥ 512	2	256	≥ 512	≥ 512	16	128	32	64	32
A201	*E. faecium* (A201R2B0)	District	Surgery	After 48 h	A1	8	4	16	8	128	256	512	512	32	2	4	512
A202	*E. faecium* (A202R2B0)	District	Surgery	After 48 h	/	≥ 512	≥ 512	4	128	64	128	≥ 512	128	≥ 512	64	128	512
	*E. faecium* (A202R3B0)			Discharge	/	8	2	≥ 512	64	128	≥ 512	≥ 512	≥ 512	128	128	≥ 512	≥ 512
A203	*E. faecalis* (A203R2B0)	District	Surgery	After 48 h	Singleton 2	≥ 512	≥ 512	4	128	≥ 512	≥ 512	≥ 512	≥ 512	≥ 512	128	≥ 512	≥ 512
A206	*E. faecium* (A206R1B0)	District	Surgery	Admission	A1	8	32	16	128	64	256	≥ 512	256	64	2	8	≥ 512
	*E. faecium* (A206R2B0)			After 48 h		8	8	16	8	64	≥ 512	≥ 512	256	32	0.5	4	128
A207	*E. faecalis* (A207R1B0)	District	Surgery	Admission	Singleton 3	16	16	4	256	128	≥ 512	≥ 512	≥ 512	128	64	32	256
A209	*E. faecium* (A209R1B0C1)	District	Surgery	Admission	A3	≥ 512	4	8	32	64	128	128	8	64	128	32	≥ 512
A210	*E. faecalis* (A210RB0C2)	District	Surgery	Admission	B2	/	32	16	2	128	256	≥ 512	≥ 512	64	256	≥ 512	512
G700	*E. faecalis* (G700R1B0C2)	Tertiary	Medicine	Admission	Singleton 4	16	4	8	256	128	512	256	≥ 512	64	32	512	256
	*E. faecalis* (G700R2B0)			After 48 h	/	≥ 512	≥ 512	2	256	64	512	≥ 512	≥ 512	128	256	≥ 512	≥ 512
G701	*E. faecalis* (G701R1B0C1)	Tertiary	Medicine	Admission	Singleton 5	16	64	≥ 512	≥ 512	≥ 512	256	≥ 512	128	32	512	≥ 512	512
	*E. faecalis* (G701R2B0C1)			After 48 h	Singleton 6	8	4	≥ 512	≥ 512	≥ 512	512	≥ 512	128	64	128	256	256
G702	*E. faecalis* (G702R1B0)	Tertiary	Medicine	Admission	Singleton 7	16	256	≥ 512	≥ 512	≥ 512	≥ 512	≥ 512	512	64	128	512	256
G802	*E. faecalis* (G802R1B0)	Tertiary	Surgery	Admission	Singleton 8	16	64	≥ 512	4	8	16	≥ 512	32	2	64	≥ 512	128
G803	*E. faecalis* (G803R1B0)	Tertiary	Surgery	Admission	Singleton 9	16	4	2	256	128	≥ 512	≥ 512	256	256	8	32	128
G805	*E. faecalis* (G805R1B0)	Tertiary	Surgery	Admission	Singleton 12	8	8	≥ 512	≥ 512	≥ 512	256	128	64	128	128	≥ 512	64
G809	*E. faecalis* (G809R1B0C2)	Tertiary	Surgery	Admission	/	≥ 512	≥ 512	16	64	64	256	256	≥ 512	16	256	≥ 512	≥ 512
G812	*E. faecalis* (G812R2B0C1)	Tertiary	Surgery	After 48 h	Singleton 13	32	8	1	64	64	256	64	2	128	2	4	128
G812	*E. faecium* (G812R3B0)	Tertiary	Surgery	Discharge	B2	32	32	0.5	128	64	256	128	2	256	4	16	128

**Table 6:** Resistance profiles and plasmids associated with vancomycin-resistant *E. faecium* and *E. faecalis* isolated from hospitalized patients

Isolate	MLST	Resistance genes															Plasmids	
		VanC-1	VanG	Vex2	Vex3	emeA	Isa	TetM	fosB	ermB	MATE	MFS	pmrA	mepA	Lde			
*E. faecalis*																		
G701R2B0C1	ST563	**+**	-	**-**	+	**-**	-	**+**	-	**+**	**+**	+	+	-	-	rep(pUB110); CDS16(pTEF3); repA2(pTEF2); rep(pKH7)		
G702R1B0	ST6	**+**	+	**+**	+	**+**	+	**+**	-	**+**	**+**	+	-	+	-	repA2(pTEF2); CDS16(pTEF3)		
***E. faecium***																		
A206R2B0	ST822	**+**	-	**+**	**+**	-	-	-	-	-	**+**	**+**	+	-	+	-		
A201R2B0	ST636	**+**	-	**+**	**+**	-	-	-	-	-	**+**	**+**	+	-	+	-		
A108R2B0	ST1386*	**+**	-	+	+	-	-	-	-	-	+	+	+	-	+	-		
A209R1B0C1	ST97	**+**	-	+	+	-	-	-	-	-	+	+	+	-	+	-		

*New ST.

### Virulence factors

WGS data revealed that *E. faecalis* strains harboured more virulence factors than *E. faecium*, with a total of 16 virulence genes for the former compared to two for the latter. The distribution of virulence genes among these isolates are presented in Table 7. Overall, all *E. faecalis* carried at least 14 virulence genes including multiple adhesins and biofilm-associated genes like [*ace* (collagen adhesin), *cad*, *cam*E, *cCF*10, *cOB*1 (sex pheromone-associated genes), ebpA/B/C (endocarditis and biofilm-associated pili), efaAfc (cell wall adhesion expressed in serum), *Elr*A (leucine-rich protein A associated with macrophage persistence), *hyl*A, *gel*E, *Srt*A (gelatinase with protease activity), *tpx*(thiol peroxidase for oxidative stress resistance), *fsr*B (*gel*E expression)]. In contrast, all *E. faecium* harboured only two virulence genes (*efaAfm* and *acm*).

**Table 7:** Virulence profiles and plasmids associated with vancomycin-resistant *E. faecium* and *E. faecalis* isolated from hospitalized patients

Isolate	MLST	Virulence genes																	Plasmids
		Ace	camE	cCF10	cOB1	ebpA	ebpB	ebpC	efaAfs	ElrA	gelE	hylA	SrtA	tpx	fsrB	hylB	acm	efaAfm	
*E. faecalis*																			
G701R2B0C1	ST563	**+**	+	**+**	+	**+**	+	**+**	+	**+**	+	**+**	+	**+**	**+**	-	-	-	rep(pUB110); CDS16(pTEF3); repA2(pTEF2); rep(pKH7); rep9b
G702R1B0	ST6	**+**	+	**+**	+	**+**	+	**+**	+	**+**	+	**+**	+	**+**	**+**	+	-	-	repA2(pTEF2); CDS16(pTEF3)
***E. faecium***																			
A206R2B0	ST822	-	-	-	-	-	-	-	-	-	-	-	-	-	-	-	+	+	-
A201R2B0	ST636	-	-	-	-	-	-	-	-	-	-	-	-	-	-	-	+	+	-
A108R2B0	ST1386*	-	-	-	-	-	-	-	-	-	-	-	-	-	-	-	+	+	-
A209R1B0C1	ST97	-	-	-	-	-	-	-	-	-	-	-	-	-	-	-	+	+	-

*New ST.

### Multi-drug resistance (MDR) efflux pumps

Seven *enterococci* isolates were subjected to WGS, of which six were MDR-E. *faecalis* and *faecium*, that carried at least two MDR efflux pump genes including MATE, MFS, *pmr*A, *mep*A, Lde. These MDR efflux pumps encode for resistance to several families of antibiotics including fluoroquinolone, tetracycline, aminoside, macrolide and glycopetides.

### Multilocus-sequence type analysis (MLST)

MLST-analyses were performed for four *E. faecium* (A206R2B0, A201R2B0, A108R2B0, A209R1B0C1) and three *E. faecalis* (G701R2B0C1, G702R1B0) strains that were selected based on their relatedness on REP-PCR (Table 5). Four single sequence types (ST) were identified among *E. faecium* in district hospital namely ST822, ST636, ST97 along with a novel ST assigned ST1386 detected in district hospital based on seven house-keeping genes including adk, atpa, ddl, gdh, gyd, psts, purk. Similarly, three different STs were observed in *E. faecalis* based on the variation amongst the seven house-keeping genes (aroe, gdh, gki, gyd, psts, xpt, yqil). Two singletons namely ST563 and ST6 were identified in tertiary hospital while ST21 was also identified in the district hospital.

Phylogenetic analysis revealed the clonal relatedness strains between hospital levels was evident, with 90,8% identity and an allelic distance of zero between G812R3B0 (ST29) and A206R2B0 (ST822) strains originating from the tertiary and district hospitals respectively (Fig. 1).

### Mobile genetic elements (MGEs) analysis

PlasmidFinder showed that only two *E. faecalis*, ST6 and ST563, isolated in the medical ward of the tertiary hospital harboured multiple plasmid replicons. The *E. faecalis* ST6 hosted CDS16(pTEF3) and repA2(pTEF2) while the *E. faecalis* ST563 carried four plasmid replicon types namely CDS16(pTEF3), repA2(pTEF2), rep(pUB110), rep(pKH7) and a single open reading frame (ORF) in contig 1183. *E. faecium* (ST29) carried two plasmid replication proteins namely repE (pAMbeta1) and, rep(pUB110) with an additional ORF in contig 287 as illustrated (Table 8).

**Table 8:** Distribution of MGEs associated with resistant*E. faecalis*and *E. faecium*

Isolates	MLST	Plasmids	IS	Transpososns and composites
*E. faecalis*				
G701R2B0C1 ***E. faecalis***	ST 563	rep(pUB110); CDS16(pTEF3); repA2(pTEF2); rep(pKH7), rep9b repUS43; repUS12;	ISLgar5; IS256; ISEnfa1	Tn 6009 Cn-5527-ISnfa1 Cn-936-ISEnfa1
G702R1B0 ***E. faecalis***	ST 6	rep(pUB110); CDS16(pTEF3); repA2(pTEF2); rep(pKH7), rep9b repUS43; repUS12;	ISLgar5; IS256; ISEnfa1	Tn 6009 Cn-5527-ISnfa1 Cn-936-ISEnfa1
***E. faecium***				
A206R2B0 ***E. faecium***	ST 822	repUS43; rep1; repUS15	IS 16; ISS1N; IS256; ISEf1	Tn6009
A901RB0 ***E. faecium***	ST 636	rep1	ISS1N; ISEf1; ISEnfa3; IS256; ISEfm1	/
A108R2B0 ***E. faecium***	ST 1386*	repUS 13; rep1	IS256; ISEfm1; IUSLgar5	/
A 209R1B0C1 ***E. faecium***	ST 97	rep1	ISS1N; IS256	/

ISFinder reveals that all isolates (*n* = 7; 100%) harbored insertion sequences and transposable elements conferring resistance to several antibiotic families. These isolates carried at least 12 insertion sequences and the most common IS family observed were IS256, IS982, IS3, IS1380, IS110, IS5, IS200/605, IS1182, IS1595. The main transposable elements observed was Tn3 family including specifically Tn1546 among all isolates.

PhasterFinder showed that all strains (100%) hosted at least one intact bacteriophage. Several prophages were identified in *E. faecalis* ST6 and PHAGE_Bacill_phBC6A52_NC_004821 was the most prevalent intact prophage followed by PHAGE_Lactob_PLE2_NC_031036 observed among *E. faecalis* and *faecium*. In addition, PHAGE_Entero_vB_EfaS_AL2_NC_042127 responsible to slide clamp DNA polymerase was especially observed among *E. faecalis* DENG1.

CRISPRFinder identified CRISPR (Clustered Regularly Interspaced Palindromic Repeats) regions were observed among all the strains. At least one CRISPR1 array was identified in these isolates. The CRISPRs were more represented in *E. faecalis* DENG1 than *E. faecium* V583. CRISPR1 and CRISPR2 were located at nucleotides 194,435 to 195,133 with 11 spacers and 203,887 to 205,203 with 21 spacers, respectively.

## Discussion

Multi-drug resistant *E. faecium* and *E. faecalis* remain an important bacterial species implicated in severe, difficult-to-treat infections globally. Hospitalized patients who were followed-up at three-time points for colonization with these bacteria showed an overall prevalence of 50%, 57% and 83% MDR-E. f*aecium* and MDR-E. *faecalis* at admission, after 48 H and at discharge respectively. These findings are higher than a South African prevalence study reported in 2000 which revealed 11% of high-risk patients colonized by MDR-*E*. *faecium* and MDR-*E*. *faecalis* [8] and generally lower than an Argentinian study that showed a 77% prevalence of MDR-*E*. *faecalis* and *faecium* from rectal swabs of hospitalized patients, with the ICU (47%) and general medicine wards (36%) being the main affected units.

Gender, antibiotic use, co-morbidity, previous hospitalization, referral from district to tertiary hospital were the main risk factors identified at admission while hospitalization in a surgical ward increased the odds of VRE colonization after 48 h. Our results are consistent with an Australian hospital-wide point prevalence study which revealed that age, duration of hospitalisation, antibiotic use and ward type were the main risk factors for MDR-*E. faecium* and MDR-*E. faecalis* colonisation in a tertiary hospital in Melbourne [17].

None of the isolates were tested positive for *Van*A and *Van*B genes but exhibited vancomycin- resistance as evident from the MICs. Although the *Van* C-1 gene is an intrinsic chromosomal gene of *E. gallinarum* and *E. casseliflavus*, its presence in our *E. faecium* could probably be attributed to horizontal gene transfer [18]. The *Van* C-1 gene was first described in vancomycin susceptible *E. faecalis* isolated from pig manure [10, 18] intimating that the chromosomal location of intrinsic resistant genes does not preclude horizontal genes tranfer to other species, therby contributing to species diversification [10, 18]. The mobility of the *Van*C-1 gene may result in laboratory misidentification of *E. gallinarum* and *E. casseliflavus* whose identification is premised on the presence of this gene. The presence of multidrug resistant efflux pumps namely MATE, MFS, *pmr*A, *mep*A, Lde harboured by all isolates could explain the high level of multi-drug resistance of our isolates [19].

The most interesting finding of the study was the likely inter-ward, inter-patient and intra-hospital spread of *E. faecalis* strains, isolated from two patients (A100R3B0 and A105R2B0) hospitalized in medical ward in the district hospital, which were closely related and shared a common ancestor with one patient (A200R3B0) from the surgical ward of the same hospital. Of note is the fact that these strains were identified at different time-points (after 48 h and discharge), confirming the dissemination of this cluster within this hospital. Similarly, *E. faecalis* also were detected in two patients (A107R1B0 and A210RB0C2) hospitalized in the medical and surgical wards in this hospital, suggesting that *E. faecalis* strains might likely circulate within wards in the district hospital and could be implicated in future nosocomial infections. The fact that all *E. faecalis* STs detected in this study carried a minimum of 14 virulence genes attests to their high pathogenicity (Table 7). The *cam*E gene encoding for sex pheromone activates the conjugation of the plasmid pAM373 that drives the transfer of virulence and resistance determinants among enterococci. Additionally, the *fsr*B gene encoding for biofilm formation was associated with ST6 and ST583 with the former isolated in the tertiary hospital and the latter in the district hospital (Table 7).

The *E. faecium* strains, A109R2B0, A201R2B0, A206R1B0 and A206R2B0 belonging to cluster A1, evidenced intra-hospital and inter-ward dissemination in the district hospital (Table 2). However, the detection of one isolate at admission and the other after 48 h intimated that they probably emerged in the community, entered the district hospital, as the first level of care, where they spread across wards (Tables 2 and 3).

**Fig. 2:**
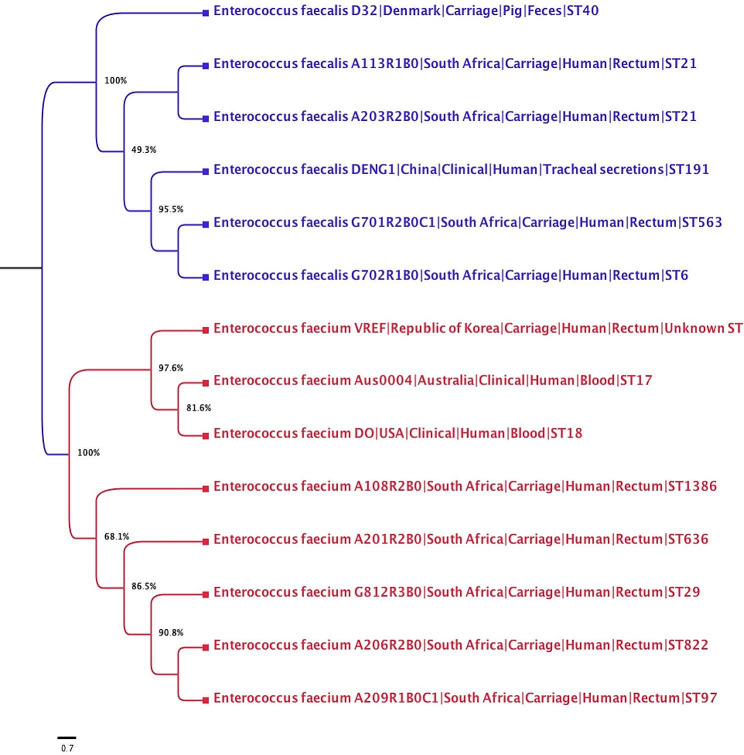
Dendograms of vancomycin-resistant *E. faecium* (A) and *E. faecalis* (B) from faecal carriage of hospitalized patients in South Africa

MLST analysis of *E. faecium* isolates confirmed a high level of genetic diversity. An interesting finding of this study was the characterization of a novel lineage *E. faecium* ST1386 (1–4–9–6–1–20–3) isolated in the rural, district hospital. In addition, the ST 822, 636, and 97 were identified in the same hospital but in different wards, suggesting that different clonal lineages of *E. faecium* are circulating in this hospital. The detection of these different STs along with two virulence genes (*acm* and *efaAfm*) suggests that various clonal lineages of vancomycin-resistant *E. faecium* are actively disseminating within the communities and could enter hospital settings where they could increasingly be associated with high mortality and morbidity rates. The scarcity of data on the population structure of these *E. faecium* STs in African countries makes it difficult to discuss the regional dissemination of these lineages detected in South Africa. Although these STs have rarely been reported in other countries to date, the variability in their allelic profiles shows high levels of diversity amongst *E. faecium*, suggesting non-human origin. This result is similar to the study of Weng et al. (2013) who demonstrated 27 pulsotypes and four STs (ST17, ST78, ST203, ST601) associated with *E. faecium* isolated from clinical samples in a tertiary teaching hospital in Malaysia [21]. Furthermore, phylogenetic analysis reveals that all *E. faecium* isolated from carriage in district hospital were closely related with 100% of similarity to *E. faecalis* isolated from clinical sample in Australia, USA and Republic of Korea respectively, suggesting the probable dissemination of *E. faecium* across the local and national levels respectively Fig. 2.

A limitation of this study was the small sample size subjected to WGS. While we postulated likely inter-ward, inter-patient and intra-hospital spread of *E. faecalis* strains, isolated from two patients we acknowledge that sharing a common ancestor is not a strong link to suggest this spread and acknowledge that supplementary analyses such as conjugation experiments and using a larger sample size and hospitals may help strengthen this association.

## Conclusion

Our study established the genetic diversity and clonal dissemination of various multi-drug resistant *E. faecalis* and *E. faecium* lineages across wards and within hospitals in uMgungundlovu district. The presence of plasmids in two *E. faecalis* and all *E. faecium* further contributed to the phenotypic and genotypic plasticity of these resistant bacteria which could be linked to easy transfer of resistance genes. Furthermore, the detection of several virulence genes and spread within and between wards and hospitals respectively could be explained the diverse originating strain from hospital environment to hospitalized patients while certify the adaptative capacity of *E. faecalis*. We report here the faecal carriage of high virulent and resistant *Enterococcus faecalis* and *faecium* among hospitalized patients in uMgungundlovu district. These isolates identified at admission and at discharged were genetically diverse and highly pathogenic. This suggests the need of real time surveillance of MDR- *E. faecium* and *E. faecalis* among hospitalized patients to identify and contain carriage and spread of these multi-drug resistant bacteria in hospitals and communities in South Africa.

### Electronic supplementary material

Below is the link to the electronic supplementary material.

Supplementary Material 1

## Data Availability

Whole genome sequencing data is available in the NCBI database under the BioProject accession PRJNA417366. The genome sequence of E. faecalis and E. faecium isolates, G701R2B0C1, G702R1B0, A206R2B0, A201R2B0, A108R2B0, and A209R1B0C1, has been deposited at DDBJ/EMBL/GenBank under accession numbers PGCW00000000, PGCV00000000, PGCU00000000, PGCT00000000, PGCS00000000, and PGCR00000000, respectively. The version described in this paper are PGCW01000000, PGCV01000000, PGCU01000000, PGCT01000000, PGCS01000000, and PGCR0000000, respectively.

## Declarations

### Authors affiliations

Antimicrobial Research Unit, School of Health Sciences, College of Health Sciences, University of KwaZulu-Natal, Durban 4000, South Africa;

Antimicrobial Resistance and Infectious Disease (ARID) Research Unit, Research Institute of Centre of Expertise and Biological Diagnostic of Cameroon (CEDBCAM-RI), Yaoundé, Cameroon;

Department of Microbiology-Haematology and Immunology, Faculty of Medicine and Pharmaceutical Sciences, University of Dschang, Dschang, Cameroon;

Reproductive, Maternal, Newborn and Child Health (ReMARCH) Research Unit, Research.

Institute of the Centre of Expertise and Biological Diagnostic of Cameroon (CEDBCAM-RI); Yaoundé, Cameroon;

Bioinformatics & Applied Machine Learning Research Unit, EDEN Biosciences Research Institute (EBRI), EDEN Foundation, Yaoundé Cameroon;

Sequencing Core Facility, National Health Laboratory Service, Johannesburg, South Africa.

## Abbreviations

MDR: multidrug resistance
VRE: Vancomycin resistant Enterococci
WGS: Whole genome sequencing
