# Supplementary material for: Genome analysis of multidrug resistant Enterococcus faecium and Enterococcus faecalis circulating among hospitalized patients in uMgungundlovu District, KwaZulu-Natal, South Africa
Source: BMC Infect Dis. 2024 Jul 4;24:671. doi: 10.1186/s12879-024-09380-3 (PMC11225414; doi:10.1186/s12879-024-09380-3)
Supplement: Supplementary file 1 — Supplementary Material 1 [file 12879_2024_9380_MOESM1_ESM.docx]

| Genes | Primer Sequence (5’-3’) | Amplicon size (bp) | Ref. |
| --- | --- | --- | --- |
| VanA | AF-GCGCGGTCCACTTGTAGATA | 314 | (13) |
|  | AR-TGAGCAACCCCCAAACAGTA |  |  |
| VanB | BF-AGACATTCCGGTCGAGGAAC | 326 |  |
|  | BR-GCTGTCAATTAGTGCGGGAA |  |  |
| VanC-1 | C1F-ATCCAAGCTATTGACCCGCT | 402 |  |
|  | C1R-TGTGGCAGGATCGTTTTCAT |  |  |
| VanC2/3 | C2F-CTAGCGCAATCGAAGCACTC | 582 |  |
|  | C2R-GTAGGAGCACTGCGGAACAA |  |  |

**Supplementary Table 1: Oligonucleotide sequences used to identify vancomycin resistance genes**
